# Risk Factors of Gestational Diabetes Mellitus Among Pregnant Women Attending Antenatal Care in King Saud Medical City, Riyadh, Saudi Arabia

**DOI:** 10.7759/cureus.67701

**Published:** 2024-08-24

**Authors:** Adeebah Mahha, Roaa I Maghrabi, Mohammed Alshuhri, Rawan I Alqurashi

**Affiliations:** 1 Family Medicine, King Saud Medical City, Riyadh, SAU; 2 Family Medicine, Prince Sattam Bin Abdulaziz University, Al-Kharj, SAU; 3 College of Medicine, Taif University, Taif, SAU

**Keywords:** saudi arabia, prevalence, risk factors, pregnancy, gestational diabetes mellitus

## Abstract

Background: Gestational diabetes mellitus (GDM) is a common prenatal condition. Many risk factors have been linked to its occurrence. This study aimed to assess the risk factors of GDM among pregnant women attending antenatal care (ANC) in King Saud Medical City (KSMC), Riyadh, Saudi Arabia, and explore the relationship between risk factors and different socio-demographic factors.

Methodology: This cross-sectional observational study was conducted on a total of 184 participants using a self-administered questionnaire distributed among pregnant women attending ANC. The collected data included sociodemographic information, medical history, obstetric history, and family history of GDM and its associated risk factors. Qualitative data was expressed in the form of numbers and percentages (N and %). The chi-square (χ^2^) test was used to examine qualitative data between two groups. The associations of GDM with these risk factors and other comorbidities were assessed, with a p-value of less than 0.05 considered significant.

Results: The prevalence of GDM was 23.9%. There was a significant association between GDM and family history of diabetes (n=39, p-value=0.0218), above normal glucose tolerance test (n=19, p-value≤0.001), and the last trimester of pregnancy (n=24, p-value=0.0139). There were no significant associations between GDM and smoking, hypertension, and adherence to health advice (p-value>0.05).

Conclusion: GDM exhibited a high prevalence among pregnant women in KSMC, Riyadh, Saudi Arabia. It showed significant associations with family history of diabetes, abnormal glucose tolerance test results, and the last trimester of pregnancy.

## Introduction

Gestational diabetes mellitus (GDM) is a medical condition that refers to intolerance of sugars or carbohydrates in the body and is identified either with the onset of pregnancy or first recognized during pregnancy. The level of intolerance of glucose in the body during GDM is still under debate, although this condition has been recognized worldwide for more than 50 years. Generally, the definition of GDM states that any level of carbohydrate intolerance that creates a hyperglycemic state in the body during the gestational state may be referred to as GDM [1,2].

GDM has been recognized as the most commonly occurring complication related to pregnancy and the prevalence of this condition has been observed to be increasing [1]. In the United States (US), it is estimated that 2-10% of pregnancies are affected by GDM. Also, women affected with GDM have an increased (35-60%) risk of developing diabetes mellitus type-2 during the later stages of their life i.e., 10-20 years after the pregnancy affected with GDM [2].

The classification of GDM is based on the nature of therapy with a positive response and describes two major types including A1GDM and A2GDM. The type of GDM that can be controlled with diet-based therapy and requires only nutritional therapy without medications is defined as A1GDM. On the other hand, A2GDM requires medicine-based intervention to achieve the required glycemic control in the body [2,3].

The major underlying mechanisms causing GDM might include delayed or absent response of pancreatic beta cells to the glycemic level in blood or insulin resistance induced by hormonal changes during pregnancy. One of the most prominent hormonal changes during pregnancy is the release of human placental lactogen hormone from the placenta. This hormone is capable of inducing metabolic changes to maintain the nutritional support of the fetus. Moreover, this hormone can also induce changes in the insulin receptors that might lead to insulin insensitivity. As a result, maternal blood sugar increases which crosses placental barriers and also stimulates the pancreatic functionalities in the fetus, and the fetal tissues might start to grow at an increased growth rate [2,4]. Also, obesity among women might induce low-grade inflammation that might lead to the production of xanthurenic acid which has been associated with the onset of GDM, prediabetes, and type 2 diabetes mellitus [2,5].

Risk factors for GDM have been described in detail to prevent the manifestation of this disease and its unwanted future outcomes. Obesity and body mass index higher than 25 kg/m^2^ have been recognized as a major risk factor for the development of GDM. Other than that, a sedentary lifestyle and lack of sufficient physical activity might increase the risk of GDM among pregnant women. Moreover, a family history of diabetes mellitus, prior history of a pregnancy with GDM, history of hypertension during pregnancy, or a previously born baby with increased weight (macrosomia) might also count as a risk factor for GDM. In addition, abnormal results of oral glucose tolerance test (OGTT) are recognized as a risk factor for GDM among pregnant women. Also, pregnancy at a later age and belonging to certain ethnicities might increase the chances of GDM development [2,6].

Furthermore, the presence of medical conditions such as cardiovascular diseases or polycystic ovarian syndrome (PCOS) might also contribute to risk factors for GDM among pregnant women. Besides, low levels of high-density lipoproteins (HDL <35 mg/dL), increased levels of triglycerides (more than 250 mg/dL), and increased levels of hemoglobin A1C (>5.7%) are considered risk factors for the development of GDM in females during pregnancy [2,6].

Preliminary interventions for the management of GDM include dietary modifications, lifestyle modifications, and regular monitoring of blood glucose levels. The dietary modifications are based on the management of caloric intake, distribution, and allotment. To ensure a physically active lifestyle, it is recommended for pregnant women to do aerobic exercises with moderate intensity for at least 30 minutes five days a week (150 minutes of exercise in one week). If glucose monitoring reveals insufficient glycemic control by dietary and exercise-based interventions, then pharmacological therapies are recommended. The baseline option for medicine-based therapy of GDM involves the use of insulin; however, some hypoglycemic agents such as metformin have also been reported for use to control GDM among pregnant women [2,7].

This study aimed to assess the risk factors of GDM among pregnant women attending ANC in KSMC, Riyadh City, Saudi Arabia, and also to explore the relationship between risk factors and different socio-demographic factors.

## Materials and methods

Study design and setting

A cross-sectional study was conducted in King Saud Medical City (KSMC), Riyadh, Saudi Arabia from May to July 2024 among the pregnant women who attended antenatal care (ANC) in KSMC. For instance, 184 pregnant women who attended ANC in KSMC were recruited.

Inclusion criteria

Pregnant women who attended ANC in KSMC during the study duration, from May to July 2024, and were aged 18 to 45 years were included in the study.

Exclusion criteria

The non-pregnant women, those aged less than 18 years or more than 45 years, attended ANC outside KSMC, and pregnant women who were already diagnosed with type 1 or type 2 diabetes were all excluded.

Data collection tools

The study was conducted using an online, self-administered questionnaire via Google Forms. The questionnaire was distributed by two of the authors, and responses were gathered online through Google Sheets. The aim of the study was clearly explained in the interface. Further, the consent form was part of the questionnaire; hence, informed consent was obtained from all the included participants. A validated questionnaire was used based on previous studies [8-10]. The questionnaire contained socio-demographic characteristics of the participant's age group, sex, nationality, and residence. The questionnaire also included questions about risk factors for GDM among pregnant women who attended ANC in KSMC, Riyadh City, Saudi Arabia. The questionnaire was translated into Arabic for easy understanding by the participants, which was then back-translated into English for data analysis.

Pilot study

The questionnaire was pretested in a pilot study over a sample of 20 participants whose results were not included in the study. Some modifications were made accordingly to ensure clarity and easy understanding of the questions.

Sampling technique and sample size calculation

A convenient non-probability sampling technique was employed to collect the data from the participants. The sample size was calculated using the EPI Info program. Based on a 95% confidence interval, a 5% margin of error, and the total population of attending women, the estimated sample size was found to be 384 and was adjusted to 422 to compensate for the 10% non-response rate.

Data collection and data analysis

Data was coded, entered, and analyzed using IBM SPSS Statistics for Windows, Version 23 (Released 2015; IBM Corp., Armonk, New York, United States). The normality of the data was tested using the Shapiro-Wilk test. Qualitative data was expressed in the form of numbers and percentages (N and %). The chi-square (χ^2^) test was used to examine qualitative data between two groups. A p-value of less than 0.05 was considered significant.

Ethical considerations

Respective approval of the study was obtained from the Research Ethics Committee in KSMC via reference number H1RI-04-May24-02. All data was kept confidential and used only for research purposes. Informed consent was obtained from all the participants before their inclusion in the study.

## Results

Out of the total 184 participants who completed the survey, 103 participants (56.0%) were in the last three months of pregnancy. Further, 140 participants (76.1%) reported no history of hypertension. Most women (n=140, 76.1%) reported not having GDM, 45 participants (24.5%) reported no family history of diabetes, and 176 participants (95.7%) reported not smoking. Additionally, 26 participants (14.1%) had glucose levels above normal, 89 participants (48.4%) reported not receiving guidance, and only 80 participants scored 5, indicating always adherence to health advice (Table 1).

**Table 1:** Descriptive statistics of participants (n=184) The data is presented as frequency (n) and percentage (%). GDM: gestational diabetes mellitus; DM: diabetes mellitus

Variable	Subgroups	N (%)
Trimester of pregnancy	First	30 (16.3)
	Second	51 (27.7)
	Third	103 (56.0)
History of hypertension	Yes	44 (23.9)
	No	140 (76.1)
Having GDM	Yes	44 (23.9)
	No	140 (76.1)
Family history of DM	Yes	131 (71.2)
	No	45 (24.5)
Smoking status	Yes	8 (4.3)
	No	176 (95.7)
Glucose tolerance test	Normal	79 (42.9)
	Above normal	26 (14.2)
	The test was not done	79 (42.9)
Guidance on GDM	Yes	90 (48.9)
	No	89 (48.4)
Adherence to health advice	1 (never)	3 (1.6)
	2	12 (6.5)
	3	41 (22.3)
	4	48 (26.1)
	5 (always)	80 (43.5)

Among those with a family history of diabetes, 39 participants (29.8%) reported having GDM. The chi-square value was 5.2646, with a p-value of 0.0218, indicating a significant association between having GDM and having a family history of diabetes (p-value<0.05) (Table 2).

**Table 2:** Association of GDM and family history of diabetes The data is presented as frequency (n). GDM: gestational diabetes mellitus

GDM	No family history	Family history	p-value
No	40	92	0.0218
Yes	5	39	

Among non-smokers, 42 participants (23.9%) reported having GDM. Among smokers, two participants (25.0%) reported having GDM. The chi-square value was 0.0, with a p-value of 1.0, indicating no significant association between having GDM and smoking status (p-value>0.05) (Table 3).

**Table 3:** Association of GDM with smoking The data is presented as frequency (n). GDM: gestational diabetes mellitus

GDM	Non-smokers	Smokers	p-value
No	134	6	1.000
Yes	42	2	

Among those with above-normal glucose levels, 19 participants (73.1%) reported having GDM. Among those with normal glucose levels, 14 participants (17.7%) reported having GDM. Among those who did not take the test, 11 participants (13.9%) reported having GDM. The chi-square value was 40.5367, with a p-value of <0.001, indicating a highly significant association between having GDM and the results of the glucose tolerance test (Table 4).

**Table 4:** Association of GDM and glucose tolerance test results among the participants The data is presented as frequency (n). GDM: gestational diabetes mellitus

GDM	Above normal	Normal	Test not done	p-value
No	7	65	68	<0.001
Yes	19	14	11	

Among those with adherence scores of 1 to 5, 1 participant (33.3%), 1 participant (7.7%), 12 participants (29.3%), 15 participants (31.3%), and 15 participants (18.8%) reported having GDM, respectively. The chi-square value was 4.9856, with a p-value of 0.2888, indicating no significant association between having GDM and adherence to health advice (p-value>0.05) (Table 5).

**Table 5:** Association of GDM with adherence to health advice level among the participants The data is presented as frequency (n). GDM: gestational diabetes mellitus

GDM	1	2	3	4	5	p-value
No	2	11	29	33	65	0.288
Yes	1	1	12	15	15	

Among those with a history of hypertension, seven participants (15.9%) reported having GDM. The chi-square value was 0.2731, with a p-value of 0.6013, indicating no significant association between having GDM and a history of hypertension (p-value>0.05) (Table 6).

**Table 6:** Association of GDM and history of hypertension among the participants The data is presented as frequency (n). GDM: gestational diabetes mellitus

GDM	No	Yes	p-value
No	124	16	0.601
Yes	37	7	

Among those in the last three months of pregnancy, 24 participants (23.3%) reported having GDM. Among those in the middle three months of pregnancy, 18 participants (35.3%) reported having GDM. Among those in the first three months of pregnancy, two participants (6.7%) reported having GDM. The chi-square value was 8.5562, with a p-value of 0.0139, indicating a significant association between having GDM and the month of pregnancy (p-value<0.05) (Table 7).

**Table 7:** Association of GDM with the month of pregnancy of the participants The data is presented as frequency (n). GDM: gestational diabetes mellitus

GDM	Last three months	Middle three months	First three months	p-value
No	79	33	28	0.0139
Yes	24	18	2	

Among those in the last three months of pregnancy, 25 participants (25.5%) reported no family history of diabetes. Among those in the middle three months of pregnancy, 11 participants (22.4%) reported no family history of diabetes. Among those in the first three months of pregnancy, nine participants (31.0%) reported no family history of diabetes. The chi-square value was 0.7060, with a p-value of 0.7026, indicating no significant association between family history of diabetes and the gestational period (p-value>0.05) (Table 8).

**Table 8:** Association of GDM with gestational period of the participants The data is presented as frequency (n). GDM: gestational diabetes mellitus

Gestational period	No GDM	Yes GDM	p-value
Last three months	79	24	0.706
Middle three months	33	18	
First three months	28	2	

Among those in the last three months of pregnancy, 100 participants (97.1%) reported not smoking. Among those in the middle three months of pregnancy, 48 participants (94.1%) reported not smoking. Among those in the first three months of pregnancy, 28 participants (93.3%) reported not smoking. The chi-square value was 1.1868, with a p-value of 0.5524, indicating no significant association between smoking status and the gestational period (p-value>0.05) (Table 9).

**Table 9:** Association of smoking status with the gestational period of participants The data is presented as frequency (n).

Gestational period	Non-smokers	Smokers	p-value
Last three months	100	3	0.552
Middle three months	48	3	
First three months	28	2	

Among those in the last three months of pregnancy, 21 participants (20.4%) had glucose levels above normal. Among those in the middle three months of pregnancy, five participants (9.8%) had glucose levels above normal. Among those in the first three months of pregnancy, none of the participants had glucose levels above normal. The chi-square value was 35.0889, with a p-value of 0.00045, indicating a highly significant association between glucose tolerance test results and the gestational period (p-value<0.001) (Table 10).

**Table 10:** Association of glucose tolerance test results and the gestational period of the participants The data is presented as frequency (n).

Gestational period	Above normal	Normal	Test not done	p-value
Last three months	21	56	26	0.000
Middle three months	5	17	29	
First three months	0	6	24	

## Discussion

GDM is a common clinical complication during pregnancy. The International Diabetes Federation expects an annual increase in the prevalence of GDM, linked to the global rise in impaired glucose tolerance, type 2 diabetes, and obesity among young adults, particularly women of reproductive age [11,12]. Several risk factors have been reported to be associated with the incidence of GDM. Thus, in this study, we assessed the risk factors of GDM among pregnant women attending ANC in KSMC, Riyadh, Saudi Arabia, and explored the relationship between risk factors and different socio-demographic factors.

The prevalence of GDM is 13.9% worldwide [13]. Several previous studies assessed the prevalence of GDM in Saudi Arabia, which ranged between 12.75% and 32.6% [10,11,14]. In the current study, 23.9% of the participants reported having GDM. Multiple predisposing factors have been associated with the development of GDM. One of the risk factors is a family history of diabetes, which represents both inherited and lifestyle elements. As previously reported by Williams et al., a family history of diabetes in the first degree relative significantly increased the risk of GDM [10]. Moreover, Alharbi et al. reported a significant association between a family history of diabetes and the incidence of GDM in pregnant women in Riyadh, Saudi Arabia [9]. Similarly, current study results showed a significant association between a family history of diabetes mellitus and the occurrence of GDM (p-value=0.0218).

Among all women (n=184), 57% (n=105) had previously undergone the glucose tolerance test, and 24.7% had above-normal glucose levels. Of these, 73.1% (n=19) eventually developed GDM, indicating a significant association between initial high glucose levels and the development of GDM (p-value≤0.001). Moreover, there was a significant association between glucose tolerance test results and the gestational period, with increased incidence of above-normal results during the third trimester of pregnancy (n=21, p-value=0.0004). High glucose levels that did not reach the GDM diagnostic thresholds were previously considered to have the same risk as normal glucose levels; however, several studies reported an increased risk of obstetric complications and a higher rate of macrosomia and large for gestational age in women with one abnormal glucose level compared to women with treated GDM [15-17].

A significant association was found between the incidence of GDM and the age of the pregnancy, with the highest incidence during the third trimester (n=24, p-value=0.0139). However, no significant difference was found between family history of diabetes and gestational age (p-value=0.05). During the third trimester, GDM was previously reported to significantly decrease the quality of life for both social life and health scales compared to uncomplicated pregnancies [18].

Prenatal smoking is among the risk factors associated with various pregnancy complications including small for gestational age, low birth weight, and preterm delivery [19]. The risk of complications is higher with a higher frequency of cigarette smoking and/or increased smoking exposure [20]. Although smoking is reported as an independent risk factor for type 2 diabetes mellitus [21,22], the data on its association with GDM is controversial with studies indicating a significant association [23,24] and several other studies reporting no significant association [25,26]. Like the latter systematic reviews and meta-analysis, no significant association between cigarette smoking and the incidence of GDM (p-value=0.05). Moreover, there was no significant association between smoking status and the gestational period (p-value=0.05).

Type 2 diabetes mellitus and hypertension commonly occur together as part of the metabolic syndrome [27]. A previous study reported that blood pressure (BP) predicted the development of incident type 2 diabetes independent of BMI and other known diabetes risk factors [28]. As previously shown by Hedderson and Ferrara, women with pre-hypertensive and hypertensive BP readings during early pregnancy were associated with an increased risk of GDM [29]. Another case-control study by Yaping et al. reported a significantly increased risk of GDM among women with hypertensive disorders of pregnancy [30]. However, current study results showed no significant association between GDM and a history of hypertension or high BP in previous pregnancies (p-value=0.05). This difference could be attributed to the different designs of studies. Moreover, no significant difference between GDM and adherence to health advice (p-value=0.05) was found, indicating more significant associations between GDM with non-modifiable risk factors.

Study limitations

The current study provided a comprehensive overview of the prevalence of GDM and the associated risk factors in pregnant women attending ANC in KSMC, Riyadh, Saudi Arabia. However, encountered several limitations that need to be addressed. First, the study was conducted in one city (Riyadh) so the generalization of the findings put it at risk of reporting bias. Second, the relatively small sample size and self-reported data put the study at risk of selection bias, response bias, and inaccuracies. Finally, given study design could not establish a causal relationship between variables.

## Conclusions

The prevalence of GDM among pregnant women in KSMC in Riyadh, Saudi Arabia, was relatively low (n=44, 23.9%). Moreover, GDM was demonstrated to have significant associations with a family history of diabetes mellitus, high blood glucose levels, and in the advanced months of pregnancy. From a public health perspective, the high prevalence of GDM in this study highlights the need for targeted screening and intervention strategies, particularly for pregnant women with a family history of diabetes or abnormal glucose tolerance. Clinically, healthcare providers should emphasize early identification and monitoring of high-risk women, especially during the last trimester, to manage and mitigate potential complications associated with GDM.
